# Accuracy of Neck stiffness, Kernig, Brudzinski, and Jolt Accentuation of Headache Signs in Early Detection of Meningitis

**Published:** 2018-01-20

**Authors:** Alireza Ala, Farzad Rahmani, Sima Abdollahi, Zahra Parsian

**Affiliations:** 1 Emergency medicine research team, Tabriz University of Medical Sciences, Tabriz, Iran.; 2 Road Traffic Injury Research Center, Tabriz University of Medical Sciences, Tabriz, Iran.

**Keywords:** Meningitis, physical examination, headache disorders, secondary, neurologic manifestations

## Abstract

**Introduction:**

The diagnostic value of clinical signs in early diagnosis of meningitis has been evaluated but the existing results are contradicting. The present study aimed to evaluate the accuracy of Kernig, Brudzinski, neck stiffness, and Jolt Accentuation of Headache (JAH) signs in this regard.

**Methods:**

In this diagnostic accuracy study, patients with suspected meningitis who were referred to the emergency department were examined regarding presence or absence of the mentioned clinical signs and screening performance characteristics of the signs were calculated. Cerebrospinal fluid analysis was used as the reference test.

**Results:**

120 cases with mean age of 48.79 ± 21.68 years (18 – 93) were studied (63.3% male). Diagnosis of meningitis was confirmed for 45 (37.5%) cases. Neck stiffness (p < 0.001), Kernig (p < 0.001), Brudzinski (p < 0.001), and JAH (p < 0.001) had significantly higher frequency among patients with meningitis. The accuracy of neck stiffness, Kernig, Brudzinski, and JAH signs in early detection of meningitis were 0.676 (95% CI: 0.575-0.776), 0.667 (95% CI: 0.552-0.782), 0.720 (95% CI: 0.619-0.821), 0.749 (95% CI: 0.659-839), respectively.

**Conclusions:**

It seems that diagnostic value of JAH is higher than other clinical signs but the accuracy of all signs is in poor to fair range. JAH had the highest sensitivity and Kernig and Brudzinski had the highest specificity.

## Introduction:

Bacterial meningitis is a prevalent disease, and its incidence in the United States is 5 to 10 cases for every 1000 population annually. This disease is particularly common amongst men (1). As a cause of mortality and morbidity, meningitis is one of 10 most fatal diseases, and results in 135000 annual deaths (2).

It is estimated that 25% of adults with bacterial meningitis and about one third of patients with tuberculosis meningitis die in spite of appropriate antibiotic treatment. One fourth of the patients who survive, suffer from constant or temporary neurologic side effects that can influence their quality of life in the future (3). Considering the dire consequences of meningitis, its diagnosis is important. Rapid and accurate evaluation of the probability of meningitis is necessary, as delayed antibiotic treatment has negative effects on the course of the disease, and can culminate in non-compensable effects on central nervous system (4-6). 

Definite diagnose is made by performing LP and analyzing cerebrospinal fluid (CSF), but this method is relatively invasive (6). Therefore, evaluation is usually initiated by physical examination and checking for presence or absence of some clinical signs such as Kernig, Brudzinski and neck stiffness. 

Another clinical sign for early diagnosis of meningitis is jolt accentuation of headache (JAH). Checking for this sign, the patient is asked to turn his head horizontally with frequency of 2-3 times in a second, and it is considered positive if the patient’s basic headache accentuated with this maneuver (7). 

The diagnostic value of the mentioned clinical signs in early diagnosis of meningitis has been evaluated in some studies but not only there is not complete information, but also the few existing results are contrary to each other (8-10). 

Based on the above-mentioned points, the present study aimed to evaluate the accuracy of Kernig, Brudzinski, neck stiffness, and JAH signs in early detection of patients suspected to having meningitis.

## Methods:

***Study design and setting***

In this diagnostic accuracy study, patients with suspected meningitis who visited the emergency department (ED) of Imam Reza Hospital of Tabriz, Iran, during a 15-month period (June 2015 to August 2016) were studied. Tabriz Imam Reza hospital is a general hospital that is affiliated to Tabriz University of medical sciences, and the admission rate of the emergency department of this hospital is 110000 annually. Written consent was taken from the patients prior to entering the study. It was explained to the patients that all of the performed tests were according to existing guidelines, and the patients won’t be deprived from routine treatments. This study was approved by the ethics committee of Tabriz University of medical sciences on 08.06.2015 under the number TBZMED.REC.1394.269.

***Participants***

Inclusion criteria were: age higher than 18, body temperature higher than 37 centigrade degrees, neck stiffness, and new onset headache. Patients with decreased level of consciousness due to any reason, patients who didn’t cooperate enough or were not able to perform the test, such as patients with vertebra osteoarthritis, muscle weakness, focal neurologic deficit and mental retardation were excluded from the study. Sampling strategy was convenient sampling.

**Table 1:** Demographic features, presenting vital signs, clinical signs and cerebrospinal fluid (CSF) analysis of the studied patients

**Variables**	**Diagnosis of meningitis**		**P **
	**Yes (n=45)**	**No (n=75)**	
**Age (year)**	42.71±21.64	52.44±21.01	0.017
**Sex **			
Male/Female ratio	29/16	47/28	1.000
**Vital signs**			
Temperature (Celsius)	38.44±0.63	37.84±0.57	˂0.001
Heart rate (/minute)	87.33±11.13	81.92±10.45	0.008
Respiratory rate (/minute)	16.00±2.41	15.39±1.93	0.128
Mean arterial pressure (mmHg)	86.07±12.71	91.72±14.76	0.035
**Clinical signs**			
Neck stiffness	29 (64.4)	22 (29.3)	˂0.001
Kernig sign	25 (55.6)	8 (10.7)	˂0.001
Brudzinski sign	24 (53.3)	7 (9.3)	˂0.001
Jolt accentuation of headache	38 (84.7)	26 (34.7)	˂0.001
**CSF analysis**			
White blood cells (/mm3)	624.38±1987.38	0.39±1.04	0.007
Protein (mg/dl)	78.00±54.93	42.03±27.44	˂0.001
Glucose (mg/dl)	64.76±37.24	77.87±32.78	0.046

Data were presented as mean ± standard deviation or frequency (%).

**Table 2:** Screening performance characteristics of different clinical signs for diagnosis of meningitis in emergency department

**Characteristics**	**Neck Stiffness**	**Kernig**	**Brudzinski**	**JAH**
True positive	29	25	24	38
False positive	22	8	7	26
True negative	53	67	68	49
False negative	16	20	21	7
Sensitivity	64.4 (48.7-77.7)	55.5 (40.1-70.0)	53.3 (38.0-68.1)	84.4 (69.9-93.0)
Specificity	70.6 (58.8-80.3)	89.3 (79.5-94.9)	90.6 (81.1-95.8)	65.3 (53.4-75.7)
PPV	56.8 (42.3-70.3)	75.7 (57.4-88.3)	77.4 (58.5-89.7)	59.3 (46.4-71.2)
NPV	76.8 (64.8-85.7)	77.0 (66.5-85.1)	76.4 (66.0-84.5)	87.5 (75.3-94.4)
PLR	1.3 (0.88-0.95)	3.12 (1.65-5.88)	3.42 (1.73-6.76)	1.46 (1.02-2.09)
NLR	0.30 (0.19-0.46)	0.29 (0.20-0.44)	0.30 (0.21-0.45)	0.14 (0.07-0.28)
J point	0.35	0.45	0.44	0.41

JAH: Jolt accentuation of headache. PPV: Positive predictive value, NPV: Negative predictive value, PLR: Positive likelihood ration, NLR: Negative likelihood ratio.

**Figure 1:**
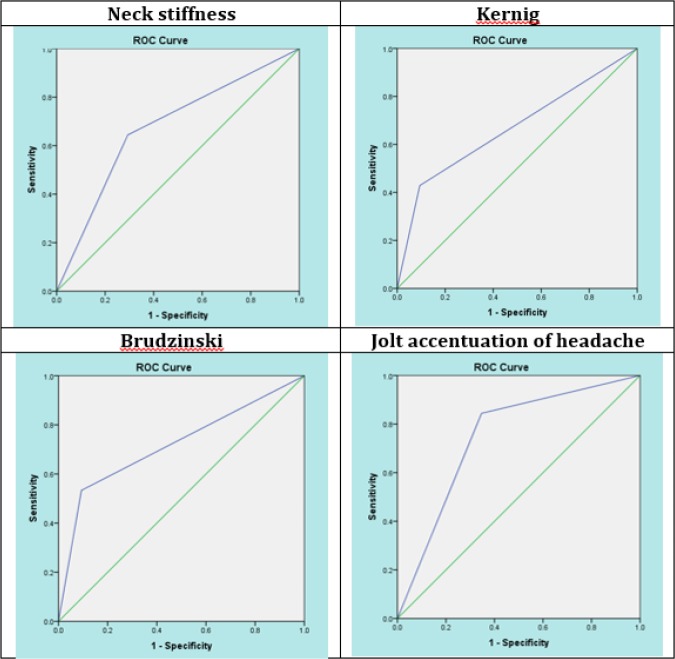
Area under the receiver operating characteristic (ROC) curve of Neck stiffness (0.676), Kerning (0.667), Brudzinski (0.720), and Jolt accentuation of headache (0.749) signs for diagnosis of meningitis in emergency department.

***Data gathering***

For all of the patients with suspected meningitis, demographic data (age, sex), vital signs, and presence of neck stiffness, Kernig, Brudzinski and JAH signs were documented. Then LP was performed and CSF was sent to the laboratory for analysis. In addition, a sample of CSF was sent for culture and another for PCR. The results of laboratory assays and physical examination were written in the pertaining check-list.

Physical examination and LP were performed by a senior resident of emergency medicine under the supervision of the attending emergency medicine physician. Laboratory reported the results without knowing the findings of physical examination. Interpreting laboratory data was done by an expert emergency physician.

***Definitions***

- Suspected meningitis cases were identified as patients with body temperature higher than 37 centigrade degrees, neck stiffness, and new onset headache.

- Existence of 5 or more white blood cells in each power field of CSF sample microscopy and CSF protein more than 45 mg/dl was considered positive for meningitis diagnosis (3, 11).

***Statistical analysis***

The sample size was calculated to be 98 using Lin Naing software, considering 95% confidence level (CI), 80% power,79% sensitivity, 82% specificity, and 12 unit difference in reported sensitivity (12). 

Data were analyzed by SPSS 17.0. Descriptive statistical methods (frequency, percentage, mean ± standard deviation) were used for describing the data. Kolmogorov–Smirnov test was used to evaluate normal distribution of the data. P value less than 0.05 was considered significant in this study. 

Screening performance characteristics (sensitivity, specificity, positive and negative like hood ratios (LR), positive and negative predictive values) of JAH test in diagnosis of meningitis were calculated (using VassarStats medical calculator) and receiver operating characteristic (ROC) curve and area under the curve (AUC) were used to measure its diagnostic accuracy. CSF analysis was considered as the reference test. AUC 90 -100 was considered as excellent accuracy, 80 -90 as good, 70 - 80 as fair, 60 - 70 as poor, and 50 - 60 as fail.

## Results:

184 patients entered the study, but 36 were eliminated from the study owing to meeting any exclusion criterion and 34 were also omitted on the grounds of lacking necessary cooperation and inaccessibility to the final diagnosis. 

Finally, 120 cases with suspected meningitis and mean age of 48.79± 21.68 years (18 – 93) were studied (63.3% male). Diagnosis of meningitis (CSF WBC>5/mm^3^ and CSF protein>45mg/dl) was confirmed for 45 (37.5%) cases. Table 1 shows the demographics, vital and clinical signs, and CSF analysis of the studied patients. Neck stiffness (p < 0.001), Kernig (p < 0.001), Brudzinski (p < 0.001), and JAH (p < 0.001) had a significantly higher frequency among patients with meningitis. Screening performance characteristics of the mentioned clinical signs are shown in table 2 and figure 2. The area under the ROC curve of neck stiffness, Kernig, Brudzinski, and JAH signs were 0.676 (95% CI: 0.575-0.776), 0.667 (95% CI: 0.552-0.782), 0.720 (95% CI: 0.619-0.821), 0.749 (95% CI: 0.659-839), respectively.

## Discussion:

The findings of the current study showed that diagnostic value of JAH in diagnosis of meningitis in emergency department is higher than other clinical signs such as Kernig, Brudzinski and neck stiffness but the accuracy of all the mentioned signs is in poor to fair range (AUC 60 to 80). JAH had the highest sensitivity (84.4%) and Kernig and Brudzinski had the highest specificity (89.3% and 90.6%, respectively) among the evaluated signs. 

In the study by Uchihara et al. that evaluated JAH test for the first time, sensitivity and specificity of the test were 97% and 60%, respectively (7). According to another study, which was performed by Aminzadeh et al. on 14 patients the sensitivity, specificity, positive predictive value, negative predictive value, positive and negative like hood ratio of JAH test were 100, 71, 78, 100, 1 and zero, respectively (11).

In the study by Nakao and et al. sensitivity of neck stiffness, Kernig, Brudzinski and JAH test for pleocytosis in CSF analysis were 13%, 2%, 2% and 21%, respectively, which were significantly lower than our findings, but the difference between the sensitivities of the tests were almost similar to our study and the sensitivity of JAH was higher than other clinical signs. The specificity of neck stiffness, Kernig, Brudzinski and JAH test in that study were 97, 98, 80 and 82 percent, respectively (13). 

In the study by Tamune and et al. the sensitivity and specificity of JAH test were 63 and 43, respectively. Contrary to our study, in their study patients with altered mental status were also evaluated (6).

Considering the results of the present and other studies absence of classic signs of meningitis is not enough for ruling out meningitis. 

Since the overall accuracy of JAH and other clinical signs is in poor to fair range, they do not have good performance alone in detection of meningitis. It seems that physicians should not solely rely on a single test or sign, and they should consider a collection of clinical signs and symptoms and history to have a better judgment. A lot of retrospective and prospective studies have shown that diagnosis of meningitis should be made according to the results of physical examination, history and CSF analysis (5, 7, 14, 15). 

***Limitation***

Small sample size and excluding the patients with lowered level of consciousness and cervical vertebral problems were some limitations of our study. 

## Conclusion:

The findings of the current study showed that diagnostic value of JAH in diagnosis of meningitis in emergency department is higher than other clinical signs such as Kernig, Brudzinski and neck stiffness, but the accuracy of all the mentioned signs is in poor to fair range. JAH had the highest sensitivity and Kernig and Brudzinski had the highest specificity among the mentioned clinical signs. 
